# New Measures, Old Conclusions: Obesity Does Not Worsen Outcomes after Elective Abdominal Aortic Aneurysm Repair

**DOI:** 10.1055/s-0042-1742699

**Published:** 2022-05-31

**Authors:** Joshua John Sommerville Wall, Katie F. Boag, Mohammed A. Waduud, Keleabetswe Pabale, Benjamin Wood, Marc Bailey, Julian A. Scott

**Affiliations:** 1 Leeds Vascular Institute, Leeds General Infirmary, Leeds, West Yorkshire, United Kingdom; 2 Leeds Teaching Hospitals NHS Trust, St James' University Hospital, Leeds, West Yorkshire, United Kingdom; 3 Faculty of Medicine and Health, University of Leeds, Leeds, United Kingdom

**Keywords:** abdominal aortic aneurysm, AAA, obesity, mortality, elective

## Abstract

**Background** The “obesity paradox,” whereby the body mass index (BMI) mortality curve is “U-shaped,” is a well-studied phenomenon in vascular surgery. However, there has been an overreliance on BMI as the measure of obesity, which has shown to poorly correlate with clinical outcomes. Robust measures such as waist-hip ratio (WHR) have been suggested as a more accurate marker reflecting central obesity.

**Objectives** The objectives of this study were to evaluate the correlation between BMI and WHR on postoperative morbidity and mortality after elective abdominal aortic aneurysm (AAA) repair.

**Methods** Data were collected from the Leeds Vascular Institute between January 2006 and December 2016. The primary outcome was mortality and secondary outcomes included length of stay (LOS) and all-cause readmission. Binary logistic regression, linear regression, and correlation analysis were used to identify associations between BMI and WHR in relation to outcome measures.

**Results** After exclusions, 432 elective AAA repairs (281 open surgical repair [OSR] and 151 endovascular aneurysm repairs [EVARs]) were identified to be eligible for the study. The combined 30-day and 4-year mortality was 1.2 and 20.1%, respectively. The 30-day readmission rate was 3.9% and the average LOS was 7.33 (standard deviation 18.5) days. BMI data was recorded for 275 patients (63.7%) and WHR for 355 patients (82.2%). Logistic regression analysis highlighted no association between BMI and WHR with mortality, readmission, or LOS following OSR or EVAR.

**Conclusion** The results of this study suggest patients should not be denied treatment for AAA based on obesity alone.

## Introduction

The “obesity paradox” has been widely studied in vascular surgery, specifically on abdominal aortic aneurysm (AAA) repair. 1 2 3 4 The paradox's main dictum is that the body mass index (BMI; mass (kg)/height (kg) 2 )-mortality curve is typically “U-shaped” with patients who are at the extremes of BMI being more likely to die. 5 6 Other studies, however, have shown no correlation between obesity (i.e., BMI > 30) and all-cause mortality; and a third group has shown decreased mortality in individuals with a raised BMI. 7 8 9 10 11

One explanation for the variance in results is the reliance on BMI to measure obesity. Data suggests individuals with central obesity defined as “ *normal* ” based on BMI had the worst long-term survival even compared with overweight and obese counterparts 12 ; owing to this and other discrepancies, there have been suggestions that BMI is suboptimal for defining obesity. 5 13

Furthermore, the World Health Organization have suggested that waist-hip ratio (WHR) may provide a more practical correlate of abdominal fat distribution and associated ill health. 14 The INTERHEART study 15 also showed that WHR was predictive of myocardial infarction whereas BMI was not. WHR has been shown to predict adverse events after elective colorectal surgery 16 ; and indeed, when compared with BMI, only measures of abdominal obesity such as WHR were found to have a significant positive association with AAA presence. 17

Given the inherent issues with BMI as a measure of central obesity, yet overreliance upon this measure widely across the literature, a comparison of BMI versus WHR in terms of predicting outcomes is required. The primary aim of this study was to compare BMI and WHR as predictors of mortality postelective AAA repair. Secondary aims looked at the independent variables ability to predict all-cause readmission and length of stay (LOS).

## Materials and Methods

### Data Collection

A review of a prospectively held local registry, the Health Quality Improvement Partnership National Vascular Registry, was undertaken to identify consecutive patients undergoing AAA repair at Leeds Vascular Institute between January 2006 and December 2016. Exclusions were made based on BMI (i.e., BMI < 18.5) owing to the scarcity of the data and on nonelective repair (i.e., urgent and emergency intervention).

### Patient Demographics and Outcome Measures

Patient demographic data collected included: age, AAA size, sex, repair type, American Society of Anesthesiologists Physical Status Classification System grade (ASA), 18 smoking history, diabetes, hypertension, ischemic heart disease (IHD), congestive heart failure (CHF), chronic kidney disease (CKD), cerebrovascular disease (CVD), and chronic obstructive pulmonary disease (COPD).

The date of death was retrieved from local electronic patient health records. Mortality was assessed at 30 days and 4 years. Similarly, the LOS and 30-day readmission were identified.

### Measurements of Adiposity

Patient BMI was measured during patient preassessment prior to intervention. WHR was obtained by analyzing preoperative routine computerized tomography (CT) imaging. The “waist” measurement was calculated as the abdominal skin circumference measured at the third lumbar vertebra at the upper end plate and the “hip” measurement was calculated using the skin circumference at the level of the tip of the greater trochanters.



**Fig. 1:**
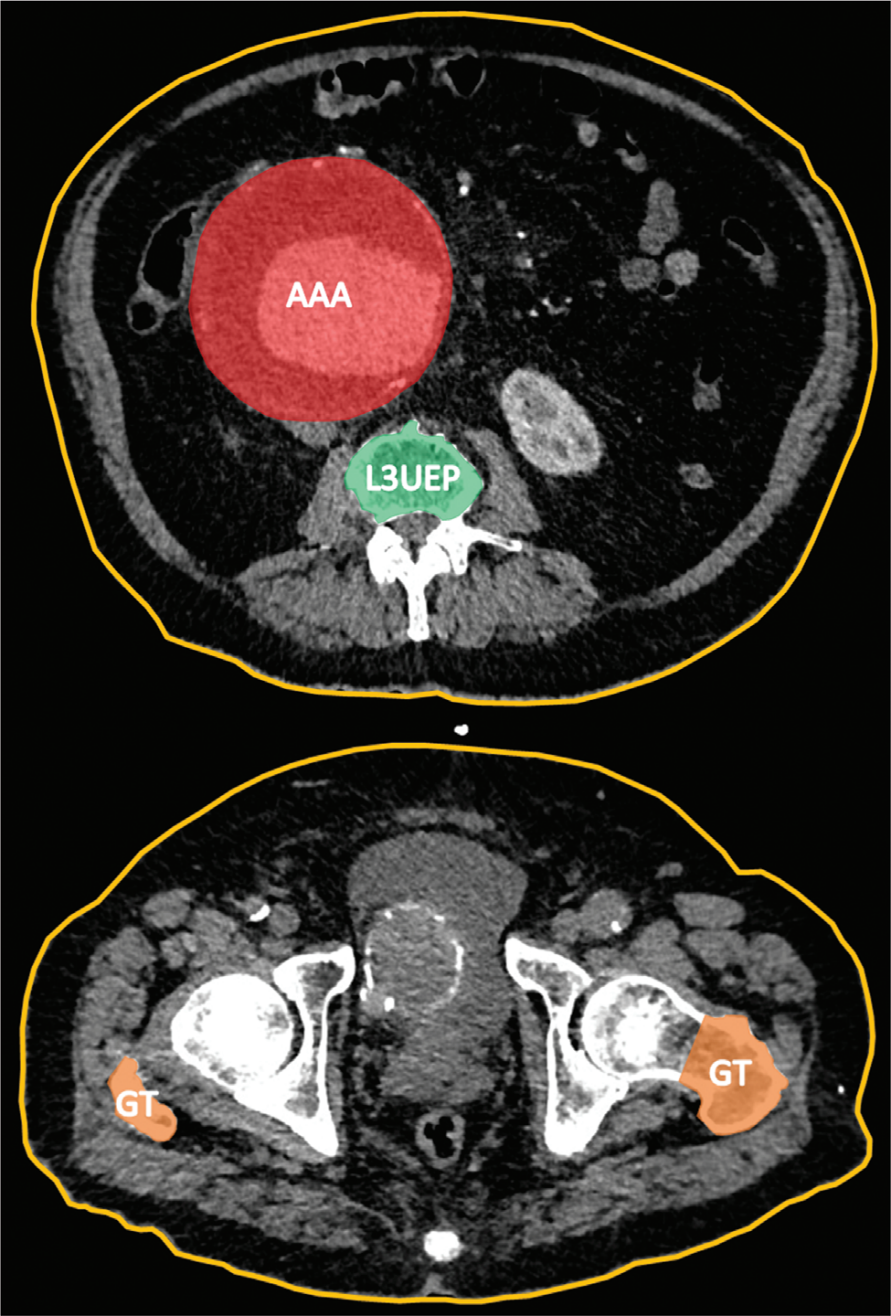
An example measurement of skin circumference (orange) at the level of the L3 upper end plate (L3UEP), and the greater trochanter (GT). Abdominal aortic aneurysm (AAA) for illustration.

CT measurements were made using a free-hand measuring tool on Picture Archiving and Communication System image viewer XERO (Agfa HealthCare, Belgium). Example measurements are shown in Fig. 1 .

### Image Analysis

All analyzed measurements were made by observer 1 (O1). O1 later repeated measurements on a random third of the cohort (O1R) and a second observer (O2) repeated measurements on a recalculated random third of the cohort. The intra- (O1 vs. O1R) and inter- (O1 vs. O2) observer differences were analyzed as mean difference ± standard deviation (SD) and a *t* -test was used to check for significant differences in measurements using techniques well described. 19 Random numbers were generated by www.random.org .

### Statistical Analysis

SPSS version 26 was used for all data analysis.

Patient demographics were described as mean ± SD for continuous data and absolute values and percentages (%) for categorical and binary data.

Adjustments were made for age, AAA size, sex, repair type, ASA, smoking history, diabetes, hypertension, IHD, CHF, CKD, CVD, and COPD. Unadjusted and adjusted binary logistic regression was used to calculate odds ratios with 95% confidence intervals describing the association between BMI and WHR in relation to 30-day and 4-year mortality and all-cause 30-day readmission. Linear regression was used to quantify the association between the independent variables and LOS.

A Bonferroni correction 20 was used to derive a *p* -value associated with statistical significance. Owing to the eight comparisons performed, a Bonferroni correction was calculated, meaning a *p* -value of less than 0.00625 (0.05/8) was required for significance. 20

## Results

A total of 681 AAA repairs were undertaken at Leeds Vascular Institute during the specified time frame. After exclusions for underweight BMI ( *n* = 5) and no urgency recorded ( *n* = 6), 670 AAA repairs remained for analysis.

**Fig. 2:**
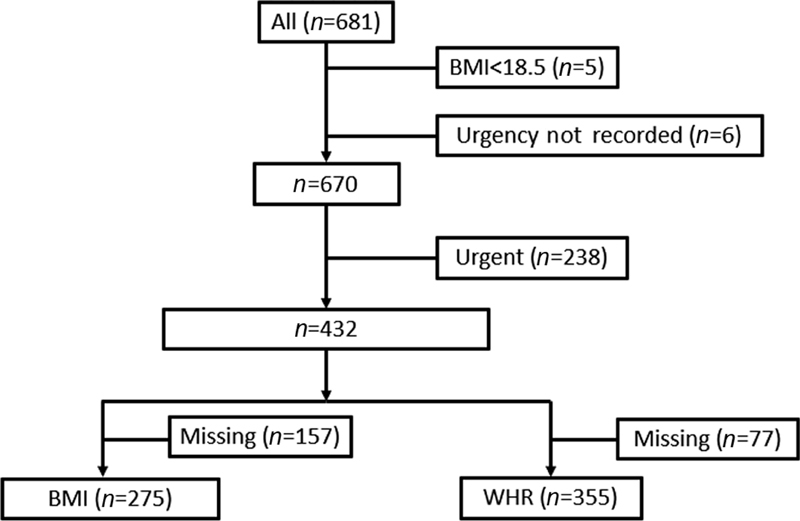
A flow diagram detailing exclusions from the study. BMI, body mass index; WHR, waist-hip ratio.

A subset of the data was identified, excluding a further 238 urgent repairs on basis of nonelective urgency level. Analysis was undertaken on the remaining 432 elective AAA repairs ( Fig. 2 ).

Baseline demographics are detailed in Table 1 . There were 5 (1.2%) deaths within 30 days, and 87 (20.1%) within 4 years. The 30-day readmission rate was 3.9% and the average LOS was 7.33 (SD 18.525) days.

**Table 1:** Baseline demographics

Variables	Mean (SD)	Missing	%
Age (y)	75 (11)	0	(0.0)
AAA size (mm)	62.37 (8.332)	31	(11.0)
BMI	28.03 (6.305)	157	(36.3)
WHR	1.025 (0.067)	77	(17.8)
LOS (d)	7.33 (18.53)	54	(12.5)
		**Count**	**%**
Gender	Men	379	87.7
	Women	53	12.2
	Missing	0	0.0
Repair type	EVAR	281	65.0
	OSR	151	35.0
	Missing	0	0.0
ASA	I	4	0.9
	II	126	29.2
	III	248	57.4
	IV	12	2.8
	V	0	0.0
	Missing	42	9.7
Smoker	Yes	288	52.8
	No	162	37.5
	Missing	42	9.7
Diabetes	Yes	48	11.1
	No	384	88.9
	Missing	0	0.0
Hypertension	Yes	150	34.7
	No	282	65.3
	Missing	0	0.0
IHD	Yes	116	26.9
	No	316	73.1
	Missing	0	0.0
CHF	Yes	13	3.0
	No	419	97.0
	Missing	0	0.0
CKD	Yes	30	6.9
	No	402	93.1
	Missing	0	0.0
CVD	Yes	16	3.7
	No	416	96.3
	Missing	0	0.0
COPD	Yes	59	13.7
	No	373	86.3
	Missing	0	0.0
30-d mortality	Yes	5	1.2
	No	427	98.8
	Missing	0	0.0
4-y mortality	Yes	87	20.1
	No	345	79.9
	Missing	0	0.0
30-d readmission	Yes	17	3.9
	No	415	96.1
	Missing	0	0.0

Abbreviations: AAA, abdominal aortic aneurysm; ASA, American Society of Anesthesiologists; BMI, body mass index; CHF, congestive heart failure; CKD, chronic kidney disease; COPD, chronic obstructive pulmonary disease; CVD, cerebrovascular disease; IHD, ischemic heart disease; LOS, length of stay; SD, standard deviation; WHR, waist-hip ratio.

Note: Mean and SD are used to describe continuous data and count and % are used for categorical and binary data.

### Observer Variation

BMI was recorded at admission for 275 (63.7%) patients and preoperative CT scans allowed for the calculation of WHR for 355 (82.2%) patients. Operator 1 calculated WHR for all 355 patients which were used in the study.

### Intraobserver Error

Prior to excluding nonelective AAA repairs, Operator 1 remeasured WHRs for a random third (284/670) of the data set. Operator 2 remeasured 183. The repeat assessments by Observer 1 did not show any significant intraobserver differences in WHR (mean difference = 0.00025, SD = 0.017362, *t* -test *p* -value = 0.845), nor did the repeat measurements made by Observer 2 (mean difference = 0.00144, SD = 0.019339, *t* -test *p* -value = 0.317).

### Patient Outcomes

The results of the unadjusted and adjusted outcomes are detailed in Table 2 .

**Table 2:** Results of the unadjusted and adjusted binary logistic regression quantifying the effects of body mass index and waist-hip ratio on 30-day mortality, 4-year mortality, and 30-day all-cause readmission with associated odds ratios, 95% CIs, and *p* -value

	Unadjusted			Adjusted		
	30-d mortality						
	OR	95% CIs	*p* -Value	OR	95% CIs	*p* -Value
BMI	0.764	0.485–1.203	0.246	0.548	0.000–> 10	0.999
WHR	0.001	0.000–417.199	0.308	0.000	0.000–> 10	0.976
	**4-y mortality**					
BMI	1.002	0.953–1.053	0.943	1.017	0.967–1.070	0.514
WHR	0.087	0.002–3.724	0.203	0.170	0.001–22.979	0.479
	**30-d readmission**					
BMI	1.010	0.941–1.083	0.789	1.009	0.925–1.101	0.833
WHR	0.002	0.000–4.341	0.113	0.000	0.000–13.149	0.129
	**LOS**					
BMI	0.121	0.001–0.241	0.047	0.111	0.020–0.202	0.017
WHR	–22.414	–55.265 to 10.438	0.180	0.940	–8.472 to 10.352	0.844

Abbreviations: BMI, body mass index; CI, confidence interval; LOS, length of stay; OR, odds ratio; WHR, waist-hip ratio.

Note: Results of the unadjusted and adjusted linear regression quantifying the effects of BMI and WHR on LOS with associated ORs, 95% CIs, and *p* -value. A *p* -value of < 0.00625 was used for significance.

After Bonferroni correction, neither BMI nor WHR was found to predict mortality, readmission, or LOS in patients undergoing elective AAA repair.

## Discussion

The primary aim of determining if 30-day and 4-year mortality were associated with BMI or WHR suggested that there was no correlation. This is in keeping with much of the literature on the subject, 21 22 23 24 25 though our study is unique in utilizing both BMI and WHR as a measure of obesity in regard to outcomes after AAA repair.

LOS and 30-day readmission were not found to be correlated with BMI or WHR. This is in keeping with findings from other authors 22 and is important considering that LOS is often the largest contributor to overall cost of intervention. 19

### Study Limitations

The findings are based from experience at a single center and with significant exclusions, though the multiple measures with consistent results lend confidence to the outcome. Of note, exclusion of underweight patients may have biased the outcome described. Underweight patients often have poorer outcomes after vascular intervention and commonly have higher rates of COPD and smoking, among other variables. 1 3 6 26 Given the small number excluded ( *n* = 5) it is unlikely that this would have significantly changed the results, and exclusion allowed for comparison targeted toward obese patients who make up a majority of the caseload.

Second, though large for a single center, the number of patients is limited in comparison with national data. Third, several patients had variables missing, in particular BMI measurements were absent for 36.3% of patients included in the study.

The relatively large number of variables (13) used in modeling, in conjunction with rare outcomes (5 deaths at 30 days) may have led to overfitting and we would welcome studies from other centers to validate the results described here.

Though we accept inherent differences in open standard repair (OSR) and endovascular repair, we considered them together owing to all patients being considered for either at presentation and the limited number of patients in the study.

### Clinical Implications

Other authors have concentrated on the effects of BMI on either OSR or endovascular repair. 2 3 10 11 21 23 25 26 In this study, we have pooled data from OSR and endovascular repair as the decision to employ either technique should be made based on numerous variables by a multidisciplinary team on a case-by-case basis. Although the results from such studies may be more useful in considering the effects of obesity on a single repair type, pooling results allows for a more robust, real-world conclusion.

The lack of negative outcomes for patients with larger BMIs or WHRs suggests that obese patients should not be excluded on the grounds of obesity alone, which is an important consideration given the propensity for obese individuals to develop AAAs. 17 Though the results published here could be accounted for by selection bias, where only obese patients who are fitter than their nonobese counterparts are considered for intervention, a larger study would be required to tease out this conclusion.

Further, better powered studies are encouraged to further quantify the link or lack thereof between central obesity as quantified by WHR and the outcomes after AAA repair. The results demonstrated here, along with the plethora of literature suggesting WHR as a superior measurer to BMI, should give confidence to potential authors.

## Conclusion

This study suggests that there is no significant correlation between obesity as measured by BMI or WHR and mortality after AAA repair in an elective setting. Furthermore, LOS and 30-day readmission were also shown to not correlate with obesity. These results, alongside much of the literature, should give confidence to obese patients who are considered good candidates for intervention and to their clinicians.
